# Effects of *Porphyromonas gingivalis* Bacteria on Inflammation, Oxidative Stress and Lipid Metabolism in Models of Obese *db*/*db* Mice and 3T3-L1 Adipose Cells

**DOI:** 10.3390/microorganisms13092074

**Published:** 2025-09-05

**Authors:** Katy Thouvenot, Fanny Le Sage, Angélique Arcambal, David Couret, Wildriss Viranaïcken, Philippe Rondeau, Olivier Meilhac, Marie-Paule Gonthier

**Affiliations:** 1 UMR 1188 Diabète Athérothrombose Thérapies Réunion Océan Indien (DéTROI), INSERM, Faculty of Health, Université de La Réunion, 97410 Saint-Pierre, La Réunion, France; katy.thouvenot@univ-reunion.fr (K.T.); f.lesage@cyroi.fr (F.L.S.); angeliquearcambal@gmail.com (A.A.); david.couret@chu-reunion.fr (D.C.); wildriss.viranaicken@univ-reunion.fr (W.V.); philippe.rondeau@univ-reunion.fr (P.R.); olivier.meilhac@inserm.fr (O.M.); 2 CHU de La Réunion, 97410 Saint-Pierre, La Réunion, France

**Keywords:** obesity, adipocytes, periodontal bacteria, lipopolysaccharides, inflammation, oxidative stress, dietary polyphenols

## Abstract

During periodontitis, *Porphyromonas gingivalis* and its lipopolysaccharides (LPS) may translocate into the bloodstream and alter adipocyte function, aggravating obesity-related disorders. This study aimed to evaluate the inflammatory and metabolic effects of *P. gingivalis* in obese *db*/*db* mice, and to decipher the molecular mechanisms targeted by *P. gingivalis* or its LPS in 3T3-L1 adipocytes. Then, we determined the ability of three major dietary polyphenols, namely caffeic acid, quercetin and epicatechin, to protect adipocytes under LPS conditions. Results show that obese mice exposed to *P. gingivalis* exhibited an altered lipid profile with higher triglyceride accumulation, an enhanced pro-inflammatory response and a reduced antioxidant SOD activity in the adipose tissue. In adipose cells, *P. gingivalis* and LPS induced the TLR2-4/MyD88/NFκB signaling pathway, and promoted IL-6 and MCP-1 secretion. Bacterial stimuli also increased ROS levels and the expression of *NOX2*, *NOX4* and *iNOS* genes, while they deregulated mRNA levels of Cu/ZnSOD, MnSOD, catalase, GPx and Nrf2. Interestingly, caffeic acid, quercetin and epicatechin protected adipose cells via antioxidant and anti-inflammatory effects. Overall, these findings show the deleterious impact of *P. gingivalis* on inflammation, oxidative stress and lipid metabolism in obese mice and adipose cells, and highlight the therapeutic potential of polyphenols in mitigating periodontal bacteria-mediated complications during obesity.

## 1. Introduction

Periodontitis is a chronic inflammatory disease associated with the development of pathogenic bacteria within the dental plaque microbiome, leading to the destruction of tooth-supporting tissues. Growing evidence highlights a causal link between periodontitis and major diseases including obesity, fatty liver disease and cardiovascular disorders [1]. A bidirectional relationship has also been established between periodontitis and type 2 diabetes mellitus (T2DM) [2]. Indeed, patients with diabetes are more susceptible to develop periodontitis, while non-diabetic individuals with periodontitis exhibit higher glycemia and increased incidence of T2DM [3,4]. Moreover, periodontitis is associated with poorer glycemic control and higher prevalence of metabolic complications in patients with T2DM [4].

One proposed mechanism linking periodontitis to systemic diseases is the translocation of periodontal bacteria and/or their components into the bloodstream. Consistently, increasing evidence reports the presence of periodontal bacteria components such as DNA and specific proteases in various extraoral locations [5,6,7,8]. Once in the blood compartment, these bacterial components can trigger chronic inflammation and metabolic disorders, causing or worsening insulin resistance. Among the major periodontal bacteria, *Porphyromonas gingivalis* has drawn particular attention due to its high pathogenicity and ability to persist within host tissues [9]. Exposure to *P. gingivalis* is associated with systemic inflammation and insulin resistance [10,11,12,13]. During obesity, the adipose tissue may play a critical role in the development of insulin resistance and diabetic status. In adipose cells, periodontal bacteria virulence factors including lipopolysaccharides (LPS) bind to the innate immunity Toll-like receptors (TLR), leading to the activation of signaling pathways involving myeloid differentiation primary response 88 (MyD88) and nuclear factor ĸappa B (NFκB) mediators [14]. This leads to the production of pro-inflammatory adipokines comprising interleukin-6 (IL-6), tumor necrosis factor α (TNFα), monocyte chemoattractant protein-1 (MCP-1), leptin and resistin which may promote insulin resistance, and conversely reduces the secretion of anti-inflammatory and insulin-sensitizing adipokines such as adiponectin [15,16]. Concomitantly, the activation of these signaling pathways induces oxidative stress by altering reactive oxygen species (ROS)-producing enzymes such as NAPDH oxidase (NOX), the antioxidant enzymes superoxidase dismutase (SOD) and catalase, and the redox-sensitive nuclear factor erythroid 2-related factor 2 (Nrf2) [17,18]. Oxidative stress further maintains inflammation and insulin resistance [15]. Previously, we demonstrated that, in comparison to *Escherichia coli* LPS inducing TLR4, *P. gingivalis* LPS lead to the activation of TLR2 and pro-inflammatory signaling pathways, associated with oxidative stress in adipose cells [19]. However, *P. gingivalis* bacteria harbors numerous virulence factors which may contribute to its pathogenicity, including fimbriae, hemagglutinins, outer membrane vesicles (OMVs) and serine/threonine proteases called gingipains [20]. Interestingly, evidence was provided for an improvement of hyperglycemia and systemic inflammation in type 2 diabetic patients following oral hygiene instructions, periodontal treatments or antibiotic adjunction [21]. In addition, dietary polyphenols have emerged as promising therapeutic agents due to their well-documented antioxidant and anti-inflammatory properties [22]. It has been reported that polyphenols are able to counteract LPS-induced oxidative stress and inflammation in adipose cells [18]. Given the effects of *P. gingivalis* on the inflammatory and metabolic disorders associated with obesity, the development of innovative therapies using antioxidant and anti-inflammatory polyphenols to reduce the dysfunction of adipocytes exposed to periodontal bacteria is of high interest.

This study aimed to evaluate the capacity of *P. gingivalis* bacteria to aggravate the deregulation of the inflammatory and metabolic profile in obese *db*/*db* mice model, and to decipher the molecular mechanisms targeted by *P. gingivalis* bacteria and related LPS in murine 3T3-L1 adipocyte model. The *db*/*db* mouse model carries a spontaneous mutation in the leptin receptor gene [23], leading to early-onset obesity, insulin resistance, hyperglycemia, dyslipidemia and adipose tissue inflammation. The *db*/*db* mice exhibit key features of human metabolic syndrome and T2DM [24], making the model particularly relevant for studying how periodontal pathogens may worsen the inflammatory and metabolic profile of susceptible hosts. At the adipose tissue level, *P. gingivalis* bacteria and LPS could affect, on the one hand, mature adipocytes responsible for fat storage, and on the other hand, preadipocytes undergoing differentiation into adipocytes. To take into account this possibility in the in vitro study, a comparative evaluation of inflammatory and metabolic markers was conducted (i) in mature adipocytes exposed to an acute 48 h treatment with bacterial stimuli or (ii) in adipocytes differentiated during a chronic 12-day treatment with bacterial stimuli. In addition, we evaluated the ability of three polyphenols commonly provided by the human diet, namely caffeic acid, quercetin and epicatechin, to improve inflammatory and metabolic response of adipose cells exposed to *P. gingivalis* LPS.

## 2. Materials and Methods

### 2.1. Animals

The experimental procedure was approved by the local Ethics Committee for animal experimentation (APAFIS#6618-2016090514008307v3) and performed according to the French and European Community Guidelines for the Use of Animals in Research (86/609/EEC and 2010/63/EU). Male heterozygous *db*/*db*^+^ and homozygous *db/db* C57BL/6 mice obtained from Charles River Laboratories (Saint-Germain Nuelles, France) were used for this study. Mice were housed in a temperature-controlled room (22 ± 2 °C) with a 12 h light-dark cycle and a relative humidity of 55 ± 10%. All mice were acclimated for a week before the experiment. During the acclimation period, all mice were fed with a standard rodent chow (SAFE, Augy, France). Food and water were available ad libitum. Twelve-week-old mice (*n* = 10 *db*/*db*^+^ and *n* = 10 *db*/*db*) were fasted overnight, weighed and then anesthetized. A cervicotomy surgery was conducted to collect blood samples from the jugular vein in all animals in order to assess glycemia, by using the OneTouch Ultra Blood Glucose Monitoring System (Lifescan, Malvern, PA, USA). Here, *db*/*db*^+^ mice were used in order to assess the presence of obesity and hyperglycemia in *db*/*db* mice, in line with our published data [25]. Then, for all the following experiments, only obese *db*/*db* mice were exposed to *P. gingivalis* or vehicle. A first *db*/*db* group (*n* = 5 mice) was injected with 50 µL of the vehicle NaCl diluted in phosphate-buffer saline (PBS, 0.9%) in the jugular vein, and used as the control group. The second *db*/*db* group (*n* = 5 mice) was intravenously injected with 50 µL of a suspension of 10^7^ colony-forming units (CFU) of *P. gingivalis* in 0.9% NaCl. The selection of this dose of bacteria was based on our published work [6] and literature studies using similar or close concentrations to evaluate the impact of *P. gingivalis* on metabolic, inflammatory and vascular markers without causing lethality [12,13,26]. After 4 h, blood samples were collected by cardiac puncture after anesthesia in EDTA tubes (BD Vacutainer, Le Pont-de-Claix, France) from all animals. Samples of subcutaneous and epididymal visceral adipose tissues, liver, heart and pancreas were excised, weighted, frozen in liquid nitrogen and stored at −80 °C until analysis.

### 2.2. Bacteria Culture

*P. gingivalis* (American Type Culture Collection ATCC-33277, Pasteur Institute Collection, Paris, France) was cultured in 2.1% mycoplasma broth base (Sigma-Aldrich, St-Louis, MO, USA) supplemented with 5 μg/mL of hemin (Sigma-Aldrich) and 1 μg/mL of menadione (Sigma-Aldrich), at 37 °C in an anaerobic environment (GENbox anaer, bioMérieux, Craponne, France). *P. gingivalis* suspensions were prepared from bacterial cultures that were at their log phase of growth. Optical density measurement at 620 nm was used to measure the concentration of bacteria. Based on standards with varying bacterial concentrations, it was determined that an optical density of 0.8 was equal to 10^9^ CFU. Bacterial suspensions of required concentration were obtained by appropriately diluting the concentrated bacterial suspension in PBS.

### 2.3. Adipose Cell Culture

Murine 3T3-L1 preadipocytes obtained from the American Type Culture Collection (ATCC-CL-973, LGC, Molsheim, France) were cultivated in Dulbecco’s modified Eagle’s medium containing 25 mM glucose (Pan Biotech, Dutscher, Brumath, France), and supplemented with 10% heat-inactivated fetal bovine serum (Pan Biotech), 5 mM L-glutamine (Pan Biotech), 2 µg/mL streptomycin (Pan Biotech) and 50 µU/mL penicillin (Pan Biotech). The cells were cultured in a humidified 5% CO_2_ atmosphere at 37 °C. For the differentiation assay, preadipocytes were seeded in 6-well plates at a density of 125 × 10^3^ cells/well in 2 mL medium and allowed to grow until confluence. Two days after confluence (day 0), preadipocytes were exposed to the culture medium containing insulin (1 µg/mL, Sigma-Aldrich), isobutyl-1-methyl-xanthine (500 µM, Sigma-Aldrich) and dexamethasone (0.25 µM, Sigma-Aldrich) until day 2. Then, for a first experiment, every two days the medium was replaced with 1 µg/mL insulin-supplemented medium, and at day 10, mature adipocytes were exposed to 10^7^ CFU of commercial heat-killed *P. gingivalis* prepared after inactivation at 85 °C for 10 min (InvivoGen, Toulouse, France) or 10 µg/mL of commercial ultrapure *P. gingivalis* LPS (Invivogen) for 48 h. This experimental condition was considered as an acute 48 h exposure to bacterial stimuli. Here, the dose of 10^7^ CFU of heat-killed *P. gingivalis* was used in accordance with the dose injected in obese *db*/*db* mice model described above. The selection of LPS dose was based on our published data [19] showing that during a dose-dependent study (1–5–10 µg/mL) on 3T3-L1 adipose cells, LPS significantly increased both IL-6 and MCP-1 secreted levels at the dose of 10 µg/mL. For the second experiment, every two days the medium was replaced with 1 µg/mL insulin-supplemented medium containing 10^7^ CFU of heat-killed *P. gingivalis* or 10 µg/mL of *P. gingivalis* LPS until day 12. This experimental condition was considered as a chronic 12-day exposure to bacterial stimuli. At the end of each experiment, cell culture media, RNA and proteins were collected and stored at −20 °C (media and proteins) or −80 °C (RNA) until analysis.

### 2.4. Assessment of Cell Viability

Adipose cells were seeded in 24-well plates (40 × 10^3^ cells/well in 500 µL medium) for 24 h. Then, the medium was removed and cells were treated with 10^7^ CFU of heat-killed *P. gingivalis* or 10 µg/mL of *P. gingivalis* LPS, in the presence or not of caffeic acid, quercetin or epicatechin (10 µM) for 48 h. The choice of this dose of polyphenols was based on the pharmacological doses broadly used in the literature and in our published studies [27,28,29]. Next, cell culture medium was removed and cells were washed once with PBS, and detached by Trypsin 0.05%-EDTA 0.02% in PBS (Pan Biotech). After centrifugation (900× *g*, 4 min, 25 °C), cell staining was achieved with Trypan Blue solution (0.4% in PBS, Pan Biotech) and cell counting performed in Malassez chamber.

### 2.5. Evaluation of Lipid Droplet Accumulation

To quantify triglyceride droplet accumulation in adipocytes exposed to *P. gingivalis* bacteria or LPS according to the conditions described above, cells were washed with PBS and fixed in 10% formaldehyde (Fisher Scientific, Illkirch, France) for 20 min. Then, cells were stained for 1 h with 0.3% (*w*/*v*) Oil Red O dye (Sigma-Aldrich) prepared in 60% (*v*/*v*) aqueous isopropanol, rinsed with water twice and photographed at 40× magnification by using inverted multichannel microscope (Nikon Instruments Inc., Melville, NY, USA). Next, the Oil Red O retained in lipid droplets was eluted with 100% isopropanol (Carlo Erba Reagents, Val-de-Reuil, France) for 15 min, and the absorbance was measured at 490 nm (FLUOstar Optima, Bmg Labtech, Ortenberg, Germany).

### 2.6. Evaluation of Triglyceride, Cholesterol and C-Reactive Protein (CRP) Levels

Triglyceride levels in plasma, liver and both subcutaneous and visceral adipose tissues were measured by using Triglyceride Quantification Colorimetric/Fluorometric kit (26-K952, Biovision, Paris, France). Cholesterol and CRP levels were evaluated in both plasma and liver by using Total Cholesterol Colorimetric/Fluorometric assay kit (26-K957, Biovision) and specific Mouse CRP ELISA kit (MCRP00, R&D systems, Minneapolis, MN, USA), respectively. Values were expressed as mg/dL plasma and mg/g tissue.

### 2.7. Protein Extraction and Quantification

Samples (100 mg) from liver and both subcutaneous and visceral adipose tissues collected from mice were homogenized in 600 µL of lysis buffer (Tris-HCl (20 mM) pH 8.5, EDTA (1 mM), Triton X-100 (0.05%), pH 7.4). Tissues were lysed twice by TissueLyser II (Qiagen, Courtaboeuf, France) with two tungsten carbide balls per tube at 30 Hz for 1 min, then centrifuged at 14,000× *g* for 10 min at 4 °C. Supernatants containing proteins were collected and stored at −80 °C until analysis. Murine 3T3-L1 cells were washed and scraped in a volume of 1 mL of PBS per well. After centrifugation at 900× *g* for 4 min at 4 °C, supernatants were removed and cell pellets resuspended in lysis buffer. Protein quantification was performed by the bicinchoninic acid assay (BCA, Sigma-Aldrich) [30], using bovine serum albumin (Sigma-Aldrich) calibration curve.

### 2.8. Quantification of Adipo-Cytokines

Samples of proteins extracted from both subcutaneous and visceral adipose tissues collected from mice, and cell culture media collected from 3T3-L1 adipocytes were analyzed by using Mouse IL-6, MCP-1, TNFα, leptin, resistin and adiponectin specific ELISA kits (IL-6, 88-7064; TNF-α, 88-7324; MCP-1, 88-7391; eBioscience, Thermofischer Scientific, Dardilly, France; ELM-Adiponectin-1, ELM-Resistin-1, RayBiotech^®^, Peachtree Corners, GA, USA; Mouse Leptin AB100718, Abcam, Cambridge, UK). Absolute values were normalized to total cellular or tissue protein content assessed by BCA assay.

### 2.9. Evaluation of SOD and Catalase Activities

Samples of proteins extracted from both subcutaneous and visceral adipose tissues collected from mice were used to evaluate total SOD and catalase activities. The total SOD activity was assessed by monitoring the rate of acetylated cytochrome c reduction by superoxide radicals generated by the xanthine/xanthine oxidase system. Measurements were performed with about 20 µg (10 µL) of tissue proteins in 170 µL of reagent buffer (xanthine oxidase, xanthine (0.5 mM), cytochrome c (0.2 mM), KH_2_PO_4_ (50 mM), EDTA (2 mM), pH 7.8) at 25 °C. Assays were monitored by spectrophotometry at 560 nm (FLUOstar Optima, Bmg Labtech). Total SOD activity was calculated using a calibration standard curve of SOD (up to 6 units/mg) and expressed as international catalytic units per µg of proteins. Catalase activity assay was carried on about 20 µg (10 µL) of tissue proteins in 150 µL of Tris-HCl (25 mM, pH 7.5). Blanks were measured at 240 nm just before adding 80 µL of H_2_O_2_ (10 mM) to start the reaction. Catalase activity was determined by measuring the absorbance at 240 nm (FLUOstar Optima, Bmg Labtech) and was calculated using a calibration standard curve of an increasing amount of catalase between 12.5 and 125 units/mL. Catalase activity was expressed as international catalytic units per µg of proteins.

### 2.10. Measurement of Intracellular ROS Levels

Intracellular ROS levels were measured by evaluating the oxidation of 2′, 7′-dichlorofluorescein diacetate (DCFH-DA), according to the method previously published [31]. Briefly, adipose cells were seeded in 96-well plates (3 × 10^3^ cells/well in 200 µL medium) and allowed to grow until confluence. Two days after confluence (day 0), preadipocytes were exposed to the culture medium containing insulin (1 µg/mL, Sigma-Aldrich), isobutyl-1-methyl-xanthine (500 µM, Sigma-Aldrich) and dexamethasone (0.25 µM, Sigma-Aldrich) until day 2. Then, every two days the medium was replaced with 1 µg/mL insulin-supplemented medium. At day 10, mature adipocytes were exposed to 10 µg/mL of ultrapure *P. gingivalis* LPS in the presence or not of caffeic acid, quercetin or epicatechin (10 µM) for 3, 6 and 48 h. Then, the medium was removed and cells were treated with 10 µM DCFH-DA prepared in PBS for 45 min in a humidified 5% CO_2_ incubator at 37 °C. Next, PBS containing DCFH-DA was replaced with PBS only and fluorescence was read at excitation and emission wavelengths of 492 and 520 nm, respectively (FLUOStar Optima, Bmg Labtech).

### 2.11. Evaluation of Gene Expression

Total RNA from 3T3-L1 adipocytes was isolated with TRIzol (Ambion, Thermo Fischer Scientific, Waltham, MA, USA). Then, 1 µg of total RNA was reverse-transcribed using Random hexamer primers (Eurogentec, Liège, Belgium). Reverse transcription-quantitative polymerase chain reaction (RT-qPCR) was performed using SYBRGreen master mix (Applied Biosystems, ThermoFisher Scientific, Waltham, MA, USA) and 20 µM primers (Eurogentec). Analysis of the expression of genes encoding TLR2, TLR4, MyD88, NFκB p65, transforming growth factor-β (TGFβ), fibronectin 1 (FN1), collagen type I alpha 1 chain (Col1a1), collagen type III alpha 1 chain (Col3a1), NOX2, NOX4, inducible nitric oxide synthase (iNOS), glutathione peroxidase (GPx), Cu/ZnSOD, MnSOD, catalase, Nrf2, CCAAT enhancer binding protein alpha (C/EBPα), peroxisome proliferator-activated receptor gamma (PPARγ), sterol regulatory element binding transcription factor 1c (SREBP1c), fatty acid synthase (FAS), lipoprotein lipase (LPL), adipose triglyceride lipase (ATGL), hormone-sensitive lipase (HSL) and glucose transporter type 4 (GLUT4) was conducted. Raw data were obtained using CFX Manager software 3.1 (BioRad, Marnes-la-Coquette, France) and *GAPDH* gene expression was used for normalization of the relative expression of target genes, according to the 2^−ΔΔCT^ method. Primer sequences are listed in Table 1.

### 2.12. Statistical Analysis

Data were expressed as means ± SEM. Statistical analysis was performed by using parametric test followed by an unpaired *t* test for comparison of data obtained from the in vivo study (*n* = 5–10 mice). Concerning the results obtained from the in vitro study, we conducted 4 independent experiments (4 different cellular passages) for the acute condition and 3 independent experiments (3 different cellular passages) for the chronic condition. One-way ANOVA with Bonferroni’s multiple comparison test was performed for analyzing data concerning more than 2 groups. Significant differences were considered for a *p* value < 0.05, according to the Program GraphPad Prism (GraphPad Software Inc., version 9, San Diego, CA, USA).

## 3. Results

To assess the obesity-related phenotype of *db*/*db* mice before bacteria injection, a comparative evaluation of total body weight, subcutaneous and visceral fat mass as well as liver weight was conducted in both homozygote *db*/*db* mice and heterozygote *db*/*db*^+^ mice (Table 2). Data show that the total body weight of *db*/*db* mice was significantly more elevated than that of *db*/*db*^+^ controls. Additionally, *db*/*db* mice exhibited higher adipose tissue and liver weights compared to *db*/*db*^+^ controls, without significant differences for organs such as the heart and pancreas. Meanwhile, fasting glycemia was significantly higher in *db*/*db* mice than in *db*/*db*^+^ animals, supporting the phenotype of *db*/*db* mice as an obese and diabetic animal model in this study. Given this phenotype, only obese *db*/*db* mice were used for the following experiments.

### 3.1. Effect of P. gingivalis Bacteria on Lipid Markers in db/db Mice

**Figure 1:**
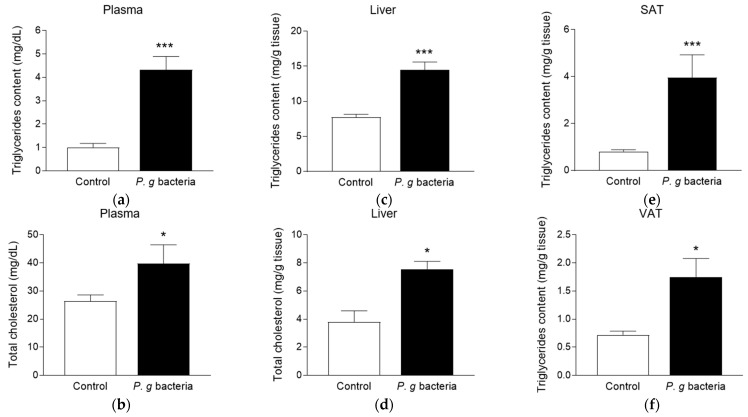
Effect of *P. gingivalis* bacteria on triglyceride and cholesterol levels in plasma, liver and adipose tissues in obese *db/db* mice. Obese *db/db* mice were intravenously injected with vehicle (Control) or *P. gingivalis* bacteria (*P. g* bacteria) at 10^7^ CFU. After 4 h, triglyceride and total cholesterol levels were measured in plasma (**a**,**b**) and liver (**c**,**d**) using colorimetric quantification kits. Triglyceride levels in subcutaneous (SAT, (**e**)) and visceral (VAT, (**f**)) adipose tissues were measured using colorimetric quantification kits. Data are expressed as mean ± SEM (*n* = 5). * *p* < 0.05, *** *p* < 0.005 as compared to Control.

To evaluate whether the exposure to *P. gingivalis* bacteria modulates lipid profile in obese mice, total cholesterol and triglyceride levels were measured in plasma, liver and adipose tissues. Bacteria-administered *db*/*db* mice were characterized by higher triglyceridemia (Figure 1a) and cholesterolemia (Figure 1b) than those measured in the control *db*/*db* mice receiving the vehicle. Hepatic levels of triglycerides (Figure 1c) and cholesterol (Figure 1d) measured in mice exposed to *P. gingivalis* were also more elevated than those determined in control animals. Moreover, we found that triglyceride contents in the subcutaneous (Figure 1e) and visceral (Figure 1f) adipose tissues were 2–4 fold higher in mice exposed to the periodontal bacteria than those detected in control mice.

### 3.2. Effect of P. gingivalis Bacteria on Inflammatory Markers in db/db Mice

**Figure 2:**
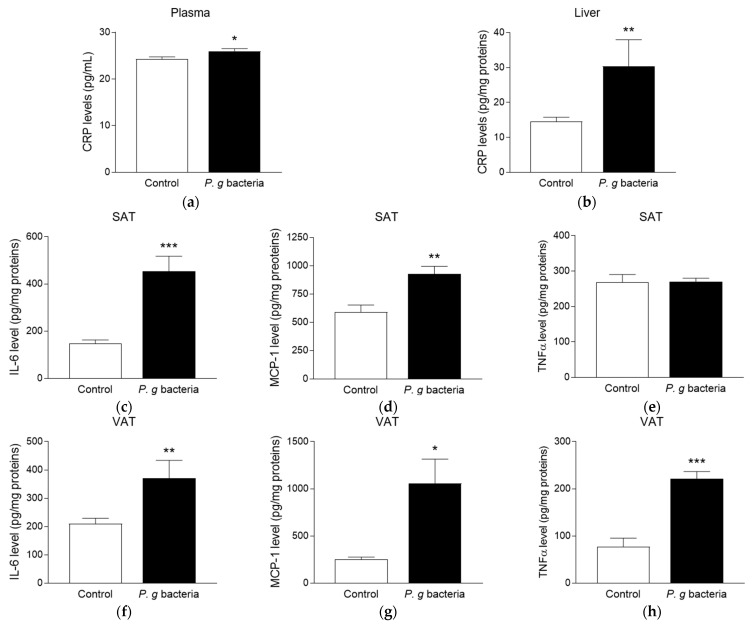
Effect of *P. gingivalis* bacteria on plasma and adipose tissue inflammatory markers in obese *db/db* mice. Obese *db/db* mice were intravenously injected with vehicle (Control) or *P. gingivalis* bacteria (*P. g* bacteria) at 10^7^ CFU. After 4 h, CRP levels in plasma (**a**) and liver (**b**) were measured by ELISA kit. Levels of IL-6, MCP-1 and TNFα were determined in subcutaneous (SAT, (**c**–**e**)) and visceral (VAT, (**f**–**h**)) adipose tissues by ELISA kits. Data are expressed as mean ± SEM (*n* = 5). * *p* < 0.05, ** *p* < 0.01, *** *p* < 0.005 as compared to Control.

To determine the impact of *P. gingivalis* bacteria on the inflammatory status of obese mice, the levels of CRP pro-inflammatory marker were measured in plasma and liver. Bacteria-administered *db*/*db* mice exhibited plasma (Figure 2a) and hepatic (Figure 2b) CRP levels significantly higher than those measured in control *db*/*db* mice injected with the vehicle. Additionally, data indicate that IL-6 (Figure 2c–f) and MCP-1 (Figure 2d–g) production was significantly increased in both subcutaneous and visceral adipose tissues from *P. gingivalis*-injected mice. Noteworthy, whereas *P. gingivalis* injection did not modulate TNFα content in the subcutaneous adipose tissue (Figure 2e), it led to a significant increase in TNFα level in the visceral adipose tissue (Figure 2h). These findings suggest the capacity of *P. gingivalis* bacteria to induce a pro-inflammatory response at the systemic, hepatic and adipose tissue levels in obese mice. 

### 3.3. Effect of P. gingivalis Bacteria on Adipose Tissue Redox Markers in db/db Mice

**Figure 3:**
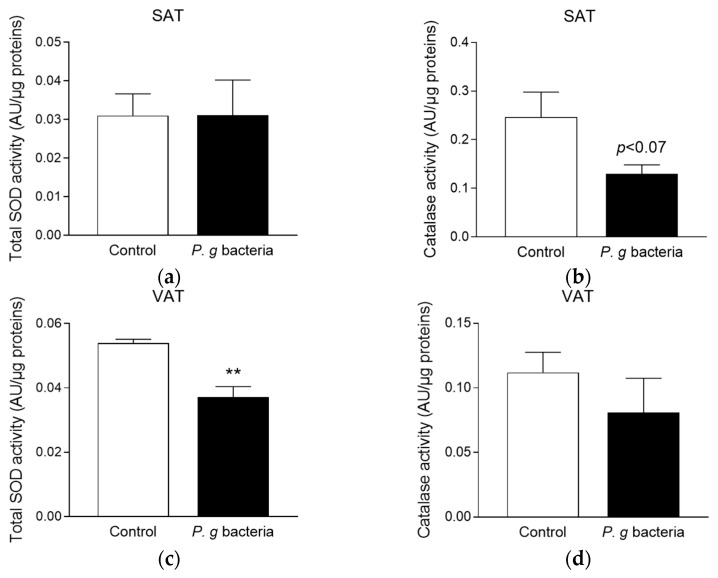
Effect of *P. gingivalis* bacteria on antioxidant enzyme activities in the adipose tissues of obese *db/db* mice. Obese *db/db* mice were intravenously injected with vehicle (Control) or *P. gingivalis* bacteria (*P. g* bacteria) at 10^7^ CFU. After 4 h, the total SOD and catalase activities were measured in both subcutaneous (SAT, (**a**,**b**)) and visceral (VAT, (**c**,**d**)) adipose tissues by fluorometric assays. Data are expressed as mean ± SEM (*n* = 5). ** *p* < 0.01 as compared to Control.

It is well established that obesity promotes oxidative stress which may contribute to adipose tissue pro-inflammatory tone and insulin resistance [15]. We evaluated the effect of *P. gingivalis* exposure on the activity of SOD and catalase antioxidant enzymes in the subcutaneous and visceral adipose tissues of *db*/*db* mice. Results show that *P. gingivalis* administration did not change the total SOD activity in the subcutaneous adipose tissue (Figure 3a). However, it led to a significant decrease in the total SOD activity in the visceral adipose tissue (Figure 3c). Catalase activity was not modulated in any location of fat deposits (Figure 3b–d), despite statistical analysis indicates a slight reduction in the subcutaneous adipose tissue (*p* < 0.07). This suggests the ability of *P. gingivalis* bacteria to alter the redox status in the adipose tissue of obese mice, in favor of oxidative stress.

### 3.4. Effect of P. gingivalis Bacteria and LPS on Adipose Cell Viability and Lipid Accumulation

**Figure 4:**
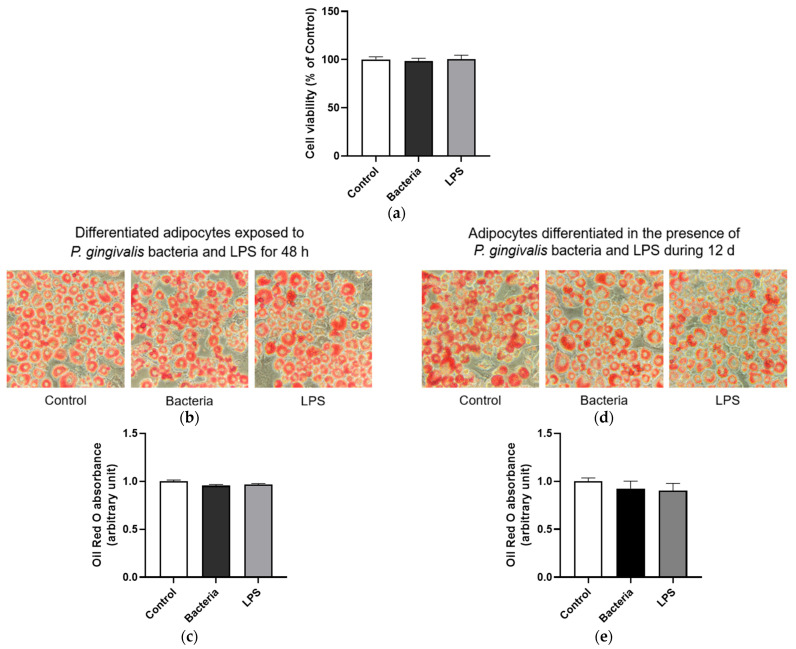
Effect of *P. gingivalis* bacteria and LPS on the viability and lipid accumulation of 3T3-L1 adipose cells. The viability of preadipocytes exposed or not to *P. gingivalis* bacteria or LPS for 48 h was measured by Trypan Blue exclusion method (**a**). Differentiated adipocytes were exposed or not to *P. gingivalis* bacteria or LPS for 48 h (**left**), and adipocytes were differentiated in the presence or not of *P. gingivalis* bacteria or LPS for 12 d (**right**). By using Oil Red O assay, lipid droplets accumulated in adipocytes were stained, visualized by microscopy (magnification 40×) (**b**–**d**), and then quantified at 490 nm (**c**–**e**). Data were expressed as mean ± SEM of *n* = 4 independent experiments for the acute condition and *n* = 3 independent experiments for the chronic condition.

To elucidate the mechanisms underlying the effects of *P. gingivalis* bacteria depicted above on the adipose tissue of obese mice, we used 3T3-L1 adipose cell model exposed either to heat-inactivated *P. gingivalis* bacteria or ultrapure LPS. Results show that the viability of adipose cells was not changed after exposure to *P. gingivalis* bacteria or LPS for 48 h (Figure 4a). To evaluate the effect of an acute or chronic treatment on lipid accumulation, differentiated adipocytes were exposed to *P. gingivalis* bacteria or LPS for 48 h or adipocytes were differentiated in the presence of bacterial components during 12 d, respectively. Data obtained by using Oil Red O assay indicate no significant changes in lipid droplet storage after an acute (Figure 4b,c) or chronic (Figure 4d,e) exposure. These data suggest that *P. gingivalis* bacteria and LPS did not modulate the viability and lipid accumulation of adipose cells in the experimental conditions tested.

### 3.5. Effect of P. gingivalis Bacteria and LPS on the Inflammatory Response of Adipocytes

**Figure 5:**
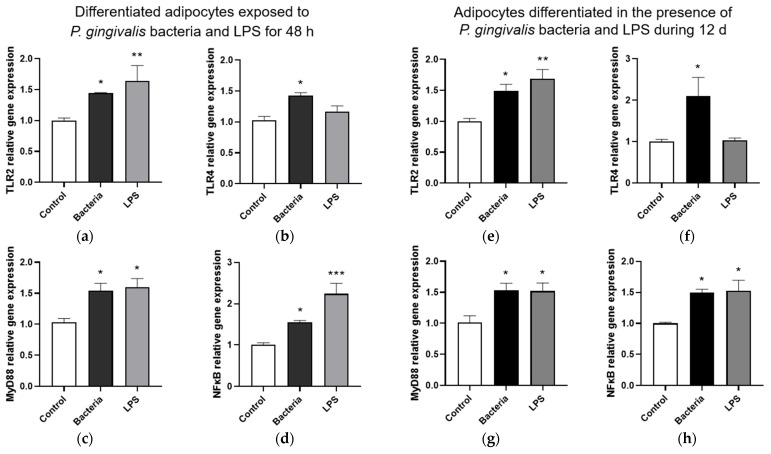
Effect of *P. gingivalis* bacteria and LPS on the inflammatory response of 3T3-L1 adipocytes. Differentiated adipocytes were or were not exposed to *P. gingivalis* bacteria or LPS for 48 h, and adipocytes were differentiated in or not in the presence of *P. gingivalis* bacteria or LPS for 12 d. The expression of genes coding for TLR2 (**a**–**e**), TLR4 (**b**–**f**), MyD88 (**c**–**g**) and NFκB (**d**–**h**) was determined by RT-qPCR and normalized to GAPDH gene expression. Data were expressed as mean ± SEM of n = 4 independent experiments for the acute condition and *n* = 3 independent experiments for the chronic condition. * *p* < 0.05, ** *p* < 0.01, *** *p* < 0.005 as compared to Control.

We measured the production of inflammatory mediators in adipocytes following an acute or chronic exposure *to P. gingivalis* bacteria and LPS. Data show that *P. gingivalis* bacteria led to an up-regulation of the expression of *TLR2* (Figure 5a–e) and *TLR4* (Figure 5b–f) genes, whereas LPS only increased *TLR2* gene expression. In addition, both bacteria and LPS induced the expression of genes coding for the adaptor protein MyD88 (Figure 5c–g) and the transcriptional factor NFκB (Figure 5d–h).

**Figure 6:**
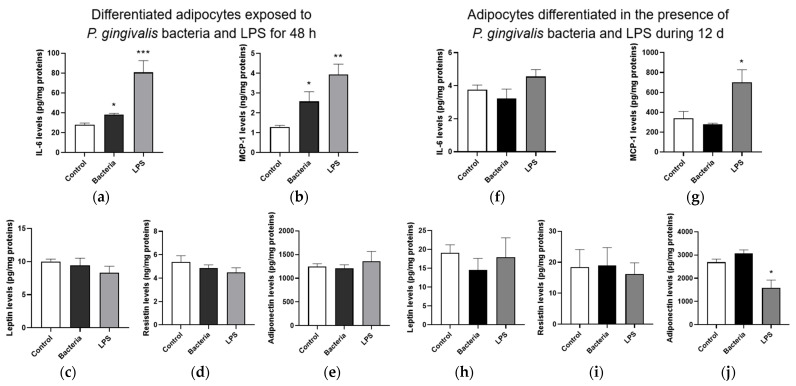
Effect of *P. gingivalis* bacteria and LPS on the secretion of adipokines from 3T3-L1 adipocytes. Differentiated adipocytes were exposed or not to *P. gingivalis* bacteria or LPS for 48 h, and adipocytes were differentiated in the presence or not of *P. gingivalis* bacteria or LPS for 12 d. The levels of IL-6 (**a**–**f**), MCP-1 (**b**–**g**), leptin (**c**–**h**), resistin (**d**–**i**) and adiponectin (**e**–**j**) secreted by adipocytes were measured by specific ELISA kits. Data were expressed as mean ± SEM of *n* = 4 independent experiments for the acute condition and *n* = 3 independent experiments for the chronic condition. * *p* < 0.05, ** *p* < 0.01, *** *p* < 0.005 as compared to Control.

Results indicate that both *P. gingivalis* bacteria and LPS also significantly increased IL-6 (Figure 6a) and MCP-1 (Figure 6b) secretion from differentiated adipocytes during an acute 48 h exposure, whereas no significant changes in secreted levels of leptin (Figure 6c), resistin (Figure 6d) and adiponectin (Figure 6e) were detected. When adipocytes were exposed to bacterial components throughout their differentiation for 12 d, *P. gingivalis* bacteria did not modulate the production of adipokines (Figure 6f–j). Importantly, LPS significantly increased MCP-1 secretion and reduced adiponectin release in chronically exposed adipocytes. These data provide evidence for the capacity of *P. gingivalis* bacteria and LPS to induce a pro-inflammatory response of adipocytes, with an extent of inflammation depending on acute or chronic exposure.

We determined whether *P. gingivalis* bacteria or LPS exposure contributes to fibrosis-associated processes in adipocytes, by evaluating the expression of genes encoding TGFβ, a key regulator of collagen synthesis, along with matrix components such as FN1, Col1a1 and Col3a1. Results indicate that a 48 h exposure of differentiated adipocytes to *P. gingivalis* bacteria and LPS did not significantly alter Col1a1 and Col3a1 mRNA levels (Table 3). However, *P. gingivalis* bacteria specifically increased *TGFβ* and *FN1* gene expression in this acute treatment condition. A similar effect on *TGFβ* and *FN1* gene expression was observed in adipocytes differentiated in the presence of *P. gingivalis* bacteria for 12 d. Furthermore, during this chronic exposure, *P. gingivalis* bacteria enhanced *Col3a1* gene expression. Of note, adipocytes differentiated in the presence of *P. gingivalis* LPS exhibited increased *FN1* gene expression, reflecting LPS action on this marker during chronic exposure. Taken together, these findings suggest that *P. gingivalis* bacteria and LPS may promote fibrosis-related processes in adipocytes.

### 3.6. Effect of P. gingivalis Bacteria and LPS on Oxidative Stress Markers in Adipocytes

Results described above show that *P. gingivalis* administration in obese *db*/*db* mice led to a reduced SOD activity in the adipose tissue. To elucidate the molecular mechanisms underlying this deleterious action of *P. gingivalis* bacteria, we assessed the expression of genes coding for redox enzymes in adipose cells. Data show that, regardless of the acute or chronic exposure conditions, both *P. gingivalis* bacteria and LPS caused an up-regulation of the expression of genes encoding the ROS-producing enzymes NOX2 and NOX4 (Table 4). Moreover, *P. gingivalis* bacteria led to an increase in iNOS mRNA levels. While only LPS enhanced *GPX* gene expression, neither *P. gingivalis* bacteria nor LPS modulated the expression of *Cu/ZnSOD* gene in adipocytes exposed to an acute treatment. Noteworthy, during a chronic exposure, the expression of *GPX* and *Cu/ZnSOD* genes was elevated by both bacteria and LPS, suggesting a time-dependent effect of bacterial stimuli.

Furthermore, an acute or chronic exposure to *P. gingivalis* bacteria and LPS raised mRNA levels of MnSOD and catalase antioxidant enzymes as well as Nrf2 redox-sensitive transcriptional factor. These results show the capacity of *P. gingivalis* bacteria and LPS to deregulate the production of markers related to oxidative stress in adipocytes.

### 3.7. Effect of P. gingivalis Bacteria and LPS on Metabolic Markers in Adipocytes

We evaluated the effects of *P. gingivalis* bacteria and LPS on the production of key adipocyte metabolic markers. These markers included C/EBPα and PPARγ related to adipogenesis, SREBP1c and FAS associated with lipogenesis, the enzymes involved in lipolysis such as LPL, ATGL and HSL, as well as the insulin-dependent glucose transporter GLUT4. Data show that an acute or chronic exposure to *P. gingivalis* bacteria and LPS did not alter the expression of genes coding for PPARγ, SREBP1c, FAS, LPL, HSL and GLUT4 (Table 5). We found that *C/EBPα* gene expression was up-regulated in adipocytes exposed to *P. gingivalis* bacteria but not LPS, during a chronic exposure. In parallel, both bacterial stimuli enhanced *ATGL* gene expression during a chronic exposure. These results suggest the capacity of *P. gingivalis* bacteria and LPS to modulate time-dependently the metabolic response of adipocytes.

### 3.8. Effect of Polyphenols on Inflammation and Oxidative Stress Markers in Adipocytes Exposed to P. gingivalis LPS

The results described above showed the impact of *P. gingivalis* bacteria and LPS on the inflammatory response and oxidative stress in adipocytes. Given that LPS are key contributors to periodontal bacteria effects, we investigated the protective roles of polyphenols in adipocytes under a 48 h cotreatment with *P. gingivalis* LPS. For this experiment, caffeic acid, quercetin and epicatechin were selected as they are major dietary polyphenols with capacities to modulate adipose tissue inflammation [22]. Data show that *P. gingivalis* LPS increased the production of all pro-inflammatory markers tested, except TLR4 and leptin, while lowering adiponectin secretion (Table 6). All polyphenols exerted anti-inflammatory effects by attenuating LPS action on TLR2/MyD88/NFκB signaling pathway mediators, without affecting the cellular viability. Quercetin and epicatechin reduced LPS-mediated IL-6 secretion, whereas only epicatechin lowered MCP-1 release. While none of the polyphenols counteracted the reduction in adiponectin secretion caused by LPS, all phenolic compounds reduced LPS-mediated resistin release. Moreover, *P. gingivalis* LPS time-dependently increased intracellular ROS levels, in line with the enhancement of the expression of genes encoding the ROS-producing enzymes NOX2 and NOX4. Interestingly, all polyphenols mitigated LPS-induced ROS elevation and changes in *NOX2* and *NOX4* gene expression. Whereas LPS did not modulate the expression of genes coding for iNOS, Cu/ZnSOD and catalase, they raised GPx, MnSOD and Nrf2 mRNA levels. Both caffeic acid and quercetin limited LPS-mediated alteration of *GPx* gene expression. Quercetin improved *MnSOD* gene expression deregulated under LPS condition. Additionally, caffeic acid, quercetin and epicatechin were able to abrogate LPS impact on *Nrf2* gene expression. Altogether, these results show the protective effects of polyphenols against *P. gingivalis* LPS action on the pro-inflammatory response and oxidative stress in adipose cells.

## 4. Discussion

This study provides evidence for the effects of *P. gingivalis* bacteria and LPS on models of obese *db*/*db* mice and 3T3-L1 adipose cells. First, our results demonstrate a significant elevation of CRP levels in both plasma and liver following *P. gingivalis* exposure in obese *db*/*db* mice. CRP is an acute-phase protein produced by the liver and is a well-established marker of systemic inflammation. Our findings are in line with literature data reporting CRP overproduction during periodontitis [32,33,34]. Elevated CRP levels are of particular concern in diabetic patients, as they are associated with a higher risk of all-cause and cardiovascular mortality [35]. It is recognized that systemic infection and inflammation affect lipid metabolism [36]. Our data show that intravenous injection of *P. gingivalis* in *db*/*db* mice significantly increased total cholesterol levels in plasma and liver, and raised triglyceride content in plasma, liver and adipose tissues. Accordingly, clinical studies reported elevated serum levels of triglycerides, total cholesterol and LDL-cholesterol in patients with periodontal infection [37,38]. Circulating levels of cholesterol are tightly regulated by LDL receptor (LDLR). The proprotein convertase subtilisin/kexin type 9 (PCSK9) acts as a key regulator of LDL-cholesterol metabolism by promoting LDLR degradation [39]. Miyazawa et al. [26] demonstrated that *P. gingivalis* upregulates PCSK9 production leading to downregulated levels of LDLR and increased systemic concentrations of total cholesterol and LDL-cholesterol in a murine model of peritoneal *P. gingivalis* infection. In parallel, Takeuchi et al. [40] identified TLR9-mediated recognition of *P. gingivalis* nucleic acids as a key mechanism driving the deleterious impact of the bacteria on cholesterol metabolism. Both the liver and adipose tissue play central roles in triglyceride storage and metabolism. Following a single administration of *Escherichia coli* LPS or cytokines in rat model, an increase in serum levels of triglycerides and VLDL can be detected within 2 h, persisting for at least 24 h [41,42]. This elevation correlates with an increase in hepatic VLDL production and impaired clearance of triglyceride-rich lipoproteins. Indeed, LPS and cytokines such as TNFα and IL-6 rapidly, within 1 h, promote de novo fatty acid and triglyceride synthesis in the liver [36]. Hardardottir et al. [43] demonstrated that during acute-phase response, LPS and cytokines reduce Apolipoprotein E production in the liver, impairing the clearance of triglyceride-rich lipoproteins. Consistently, Arimatsu et al. [12] reported that *P. gingivalis* administration in mice increases triglyceride accumulation in the epididymal adipose tissue. Noteworthy, these literature data obtained from murine or rat models different from the obese and diabetic *db*/*db* mice model used in our present study, suggest that bacteria such as *P. gingivalis* or *E. coli* may modulate the metabolism of lipids including triglycerides via signaling pathways mainly controlling inflammation during the infection [36] and not via specific mechanisms related to obesity. In our study, main adipogenesis, lipogenesis and lipolysis-related markers were not significantly altered in mature adipocytes exposed to *P. gingivalis* bacteria or its LPS, except for C/EBPα adipogenic factor slightly enhanced under chronic bacteria condition. This raises the possibility that the observed effect in *db*/*db* mice may result from systemic changes in the adipose tissue environment, which becomes enriched in triglycerides. Noteworthy, Singh et al. [44] reported that differentiating 3T3-L1 adipocytes in the presence of *P. gingivalis* for 8 d leads to an increase in the production of adipogenic factors such as C/EBPα, PPARγ and FAS. Accordingly, our data indicate an increase in C/EBPα gene expression in adipocytes differentiated in the presence of *P. gingivalis*, although other adipogenic markers remain unchanged. In agreement with Singh et al., we also found that *P. gingivalis* bacteria and LPS did not modulate adipocyte differentiation, despite they activate NFκB pathway. This result contrasts with findings showing that *E. coli* LPS led to an inhibition of adipogenesis via NFκB activation [45]. Additionally, our data show that a chronic exposure of adipocytes to *P. gingivalis* bacteria and LPS increased the expression of the gene encoding ATGL which is the enzyme catalyzing the first step of lipolysis in adipose cells. These results further emphasize the ability of *P. gingivalis* to modulate lipid metabolism, suggesting its potential role in adipose tissue remodeling. Given the key role of preadipocytes in the adipose tissue development, it would be of interest to assess the possible direct impact of *P. gingivalis* bacteria on preadipocyte proliferation rate and contribution to adipose tissue expansion.

Our data show that *P. gingivalis* administration in obese *db*/*db* mice led to an increase in the levels of IL-6 and MCP-1 in both subcutaneous and visceral adipose tissues, while TNFα levels were elevated exclusively in the visceral adipose tissue. Consistently, in *db*/*db* mice, Sharma et al. [46] reported that an intraperitoneal injection of *E. coli* LPS triggered a peak in TNFα expression in adipose tissue within 1 h, followed by a peak in IL-6 and MCP-1 levels at 4 h. This was associated with a raised production of the Suppressor Of Cytokine Signaling 3 (SOCS3), a key inhibitor of insulin signaling [47]. Similarly, other in vivo studies demonstrated that chronic exposure to *P. gingivalis* contributes to adipose tissue inflammation [10,11,12]. The overproduction of these cytokines in the adipose tissue may play a critical role in worsening insulin resistance during periodontal infection. In line with in vivo results, our in vitro data confirmed that *P. gingivalis* exposure induces an elevation of IL-6 and MCP-1 release from adipocytes. Notably, we found that both *P. gingivalis* and LPS enhanced TLR2 production, whereas only whole bacteria triggered TLR4 activation. This result is in agreement with our previous findings showing that, unlike *E. coli* LPS, *P. gingivalis* LPS primarily signals through TLR2 [19]. According to Darveau et al. [48], *P. gingivalis* produces two structurally dominant forms of LPS, differing in the acylation status of lipid A. While both forms engage TLR2 and TLR4, one preferentially binds to TLR2, whereas the other has a stronger affinity for TLR4 [49]. One limitation of the present study is that the structural forms and concentrations of LPS provided by the heat-killed *P. gingivalis* commercial solution used were not determined. Moreover, the activation of TLRs in response to whole bacteria may involve additional bacterial components such as DNA, flagellin, lipoproteins and peptidoglycan [12]. Otherwise, *P. gingivalis* is known to secrete specific gingipain proteases that inactivate pro-inflammatory mediators and enable the bacteria to evade innate immunity [50]. The presence of gingipains in the solution of heat-killed *P. gingivalis* used in the present study was not investigated and may partially explain why we did not depict significant changes in IL-6 and MCP-1 secretion during chronic exposure throughout adipocyte differentiation. Literature data reported that after 12 d of differentiation in the presence of *P. gingivalis* LPS, secreted levels of adiponectin, a key insulin-sensitizing adipokine, were significantly reduced [44]. Here, a similar reduction in adiponectin release was observed in adipocytes exposed to a chronic but not an acute LPS condition, highlighting a time-dependent deleterious effect of LPS. It will be interesting to assess molecular players of insulin-mediated signaling pathway and glucose uptake in adipocytes exposed to bacterial stimuli.

During obesity, adipose tissue fibrosis contributes to the loss of tissue plasticity, impairing fat storage and release. This, in turn, may exacerbate inflammation and promote insulin resistance, reinforcing the detrimental impact of *P. gingivalis*. We found that *P. gingivalis* bacteria specifically caused an upregulation of the expression of genes encoding TGFβ and FN1 under an acute 48 h exposure. Seki et al. [51] found that TLR4, but not TLR2, is required for LPS-mediated hepatic fibrogenesis, highlighting the role of TLR4 signaling in fibrotic processes. In the adipose tissue, TGFβ can be secreted by both adipocytes and cells of the stromal vascular fraction [52], and is known to promote the production of FN1 which is a major extracellular matrix component. Vila et al. [53] established a strong link between TLR4 activation and TGFβ1-mediated adipose tissue fibrosis. Furthermore, TGFβ signaling is known to regulate the production of pro-fibrotic factors, including collagens [54]. In agreement, our results show an increase in the expression of the gene encoding type III collagen in adipocytes differentiated in the presence of *P. gingivalis* bacteria.

Oxidative stress is known to promote the production of pro-inflammatory adipokines and to participate to obesity-related insulin resistance [15]. Our data show that after *P. gingivalis* bacteria challenge, SOD activity was decreased in the visceral adipose tissue, while only a similar trend was observed for the catalase activity (*p* < 0.07) in the subcutaneous adipose tissue of *db*/*db* mice. These findings are in agreement with data from clinical studies reporting reduced antioxidant enzyme activity in serum and saliva during periodontitis [55,56]. This highlights the ability of the periodontal bacteria to induce oxidative stress, by impairing the antioxidant defense system. Consistently, our in vitro data demonstrate that *P. gingivalis* bacteria and LPS triggered oxidative stress in adipocytes. We depicted a significant increase in mRNA levels of NOX2 and NOX4, two key ROS-producing enzymes, with a 2-fold upregulation in adipocytes differentiated under a chronic exposure to *P. gingivalis* bacteria or LPS. This is in agreement with data showing that *P. gingivalis* LPS treatment led to an elevation of mitochondrial ROS levels and the production of inflammatory cytokines in human gingival fibroblasts [57], and indicating that *P. gingivalis* impact may not be specific to adipocytes. In mammalian cells, ROS are detoxified by antioxidant enzymes including Cu/ZnSOD, MnSOD and catalase. Our data show that *Cu/ZnSOD* gene expression was increased only when adipocytes were exposed to *P. gingivalis* bacteria and LPS during chronic treatment, whereas *MnSOD*, *catalase* and *GPx* genes were upregulated in both acute and chronic exposure conditions. The discrepancy between the elevation of SOD mRNA levels in adipocytes chronically exposed to *P. gingivalis* during 12 d and the reduction in SOD enzymatic activity in the adipose tissue of obese *db*/*db* mice injected with *P. gingivalis* during an acute period of 4 h, warrants mention. It is known that mRNA levels are time-dependently regulated and do not always reflect protein abundance or enzymatic activity, due to post-transcriptional and/or post-translational regulatory mechanisms [58]. Moreover, whole adipose tissue is a complex and heterogeneous organ, comprising not only adipocytes but also various other cellular types, including immune cells such as macrophages, whose infiltration markedly increases during obesity [59]. These immune cells significantly contribute to the tissue’s overall antioxidant response and redox enzyme activities [15]. In contrast, adipocyte monocultures lack these cellular interactions and immune-derived signals, which may explain the apparent discrepancy observed between in vivo and in vitro findings. It would be of high interest to compare the activities of redox enzymes such as SOD in in vitro adipocytes and macrophages exposed to *P. gingivalis* in order to evaluate their contribution to the antioxidant response of the whole adipose tissue of *db/db* mice injected with *P. gingivalis*. Meanwhile, we found an increase in the expression of the gene encoding the redox-sensitive transcriptional factor *Nrf2* in adipocytes exposed to *P. gingivalis* bacteria and LPS. Nrf2 is established as a crucial regulator of antioxidant enzyme production. Literature data have reported that in *P. gingivalis* LPS-stimulated macrophages, NFκB/MAPK signaling pathway activation leads to nuclear accumulation of Nrf2 during oxidative stress [60]. This enhancement of the antioxidant defense system may help cells to improve their immune response against the deleterious action of bacterial components. Here, our data show that *P. gingivalis* bacteria specifically caused an increase in the production of *iNOS*, which is also a key signaling enzyme involved in oxidative stress. Such an effect of *P. gingivalis* bacteria is not specific to adipocytes as it was previously detected in immune and non-immune cells like human umbilical endothelial cells [61]. In a model of 3T3-L1 adipocytes, Singh et al. [44] demonstrated that *P. gingivalis* bacteria increased iNOS production, resulting in increased intracellular ROS production. iNOS activation was associated with a reduction in the production of MnSOD, heme oxygenase-1 (HO-1) and peroxisome proliferator-activated receptor-gamma coactivator-1alpha (PGC-1α). In parallel, the suppression of HO-1 and PGC-1α production in the adipose tissue was found to promote mitochondrial dysfunction and increased inflammation [62]. Importantly, genetic or pharmacological invalidation of iNOS was reported to exert a protective action against LPS-induced insulin resistance in mice [63].

Given the effects of *P. gingivalis* bacteria and LPS on inflammatory and oxidative stress markers in adipocytes, we evaluated the capacity of major dietary polyphenols to exert protective roles. The dose of 10 µM of polyphenols used is in accordance with the pharmacological doses broadly used in the literature and in our published studies [27,28,29]. This dose is close to plasma concentrations reaching less than 10 µM in nutritional situations, keeping in mind that polyphenols are poorly absorbed by the intestinal tract and that their metabolic fate depends on their structure and microbial catabolism [64]. Of note, our present data show that polyphenols used at 10 µM did not alter the viability of 3T3-L1 adipose cells after 48 h of treatment. Thus, the effects of polyphenols we observed here, could not be associated with a cytotoxic action. Our results demonstrate that caffeic acid, quercetin and epicatechin mitigated the inflammatory response induced by *P. gingivalis* LPS by downregulating *TLR2*, *MyD88* and *NFκB* gene expression. Moreover, quercetin reduced the secretion of IL-6, and epicatechin lowered the release of both IL-6 and MCP-1. All three polyphenols also attenuated LPS-mediated resistin secretion. These findings corroborate our published data showing the anti-inflammatory effects of polyphenol-rich extracts from medicinal plants in adipocytes exposed to *E. coli* and *P. gingivalis* LPS [18,27]. In the present study, caffeic acid, quercetin and epicatechin exerted antioxidant effects by improving the production of ROS and redox enzymes deregulated by *P. gingivalis* LPS. The antioxidant properties of polyphenols may result from multiple mechanisms comprising a direct effect on ROS-scavenging and neutralization, or an indirect action via inhibition of ROS genesis through the regulation of signaling pathways related to oxidative stress [65]. The results obtained here in mature adipocytes further support our previous findings demonstrating the protective effects of different chemical families of polyphenols against intracellular ROS accumulation in preadipocytes exposed to oxidative stress [66]. In addition, our present data show that all polyphenols suppressed LPS-induced production of NOX2 and NOX4, recognized as major ROS-producing enzymes. This raises the possibility that, by limiting ROS production, polyphenols could lower the level of antioxidant defense needed to counteract oxidative stress, as shown by the caffeic acid- and quercetin-mediated decrease in *GPx* and *MnSOD* gene expression in adipocytes under LPS condition. Given that the protective effects of polyphenols depended on the nature of the molecules considered, it will be relevant to elucidate their modality of accessibility to adipocytes and their capacity to modulate specific or common signaling pathways.

## 5. Conclusions

This study highlights the effects of *P. gingivalis* bacteria on inflammation and oxidative stress, in particular in the adipose tissue, in the model of obese *db*/*db* mice. The mechanistic study conducted on 3T3-L1 adipocyte model exposed to *P. gingivalis* bacteria and LPS led to identify underlying mechanisms that involve TLR-mediated NFκB activation and redox factors related to oxidative stress. In addition, this study demonstrates a significant impact of *P. gingivalis* bacteria exposure on lipid metabolism in *db*/*db* mice, further emphasizing the causal roles of periodontal bacteria in metabolic dysregulations during obesity. Our results provide evidence that *P. gingivalis* LPS constitute key bacterial components driving inflammation and metabolic disorders in adipocytes. However, we also observed specific alterations induced by whole bacteria, suggesting that other bacterial components contribute to these effects. Further work is needed to better understand the contribution of such components to the deleterious action of *P. gingivalis* bacteria in adipocytes. Interestingly, this study underlines the protective effects of polyphenols in attenuating *P. gingivalis* LPS-induced inflammation and oxidative stress in adipocytes. Thus, it will be of high interest to evaluate the benefits of therapeutic strategies using polyphenols to limit inflammatory and metabolic complications caused by periodontal bacteria in the context of obesity.

## Figures and Tables

**Table 1:** Primers used for RT-qPCR analysis.

Gene	Forward Sequence	Reverse Sequence
*ATGL*	CAC-TTT-AGC-TCC-AAG-GAT-GA	TGG-TTC-AGT-AGG-CCA-TTC-CT
*Catalase*	CCT-CCT-CGT-TCA-GGA-TGT-GGT-T	CGA-GGG-TCA-CGA-ACT-GTG-TCA-G
*C/EBPα*	GAG-CCG-AGA-TAA-AGC-CAA-AC	GCG-CAG-GCG-GTC-ATT-G
*Col1a1*	CATAAA-GGG-TCA-TCG-TGG-CT	TTG-AGT-CCG-TCT-TTG-CCA-G
*Col3a1*	GAA-GTC-TCT-GAA-GCT-GAT-GGG	TTG-CCT-TGC-GTG-TTT-GAT-ATT-C
*Cu/ZnSOD*	GCA-GGG-AAC-ACT-CCA-CTT	ATG-AAC-CTC-TGG-ACC-CGT
*FAS*	ACT-CCA-CAG-GTG-GGA-ACA-AG	CCC-TTG-ATG-AAG-AGG-GAT-CA
*FN1*	CTT-TGG-CAG-TGG-TCA-TTT-CAG	ATT-CTC-CCT-TTC-CAT-TCC-CG
*GAPDH*	CTT-TGT-CAA-GCT-CAT-TTC-CTG-G	TCT-TGC-TCA-GTG-TCC-TTG-C
*GLUT4*	TGC-TGG-GCA-CAG-CTA-CCC	CGG-TCA-GGC-GCT-TTA-GAC
*GPx*	TGC-TCA-TTG-AGA-ATG-TCG-CGT-CTC	AGG-CAT-TCC-GCA-GGA-AGG-TAA-AGA
*HSL*	TTC-GCC-ATA-GAC-CCA-GAG-TT	TGT-GCC-AAG-GGA-GGT-GAG-AT
*iNOS*	GCA-GCC-TGT-GAG-ACC-TTT-G	GCA-TTG-GAA-GTG-AAG-CGT-TTC
*MnSOD*	ATG-TTG-TGT-CGG-GCG-GCG	AGG-TAG-TAA-GCG-TGC-TCC-CAC-ACG
*MyD88*	TCG-AGT-TTG-TGC-AGG-AGA-TG	AGG-CTG-AGT-GCA-AAC-TTG-GT
*NFκB*	GTG-ATG-GGC-CTT-CAC-ACA-CA	CAT-TTG-AAC-ACT-GCT-TTG-ACT-CAC-T
*NOX2*	ACC-TTA-CTG-GCT-GGG-ATG-AA	TGC-AAT-GGT-CTT-GAA-CTC-GT
*NOX4*	GAT-CAC-AGA-AGG-TCC-CTA-GCA-G	GTT-GAG-GGC-ATT-CAC-CAA-GT
*Nrf2*	TTG-GCA-GAG-ACA-TTC-CCA-T	GCT-GCC-ACC-GTC-ACT-GGG
*LPL*	CCA-CAG-CAG-CAA-GAC-CTT-C	AGG-GGC-GGC-CAC-AAG-TTT-G
*PPARγ*	AAA-CTC-TGG-GAG-ATT-CTC-CT	TGG-CAT-CTC-TGT-GTC-AAC
*SREBP1c*	GAT-CAA-AGA-GGA-GCC-AGT-GC	TAG-ATG-GTG-GCT-GCT-GAG-TG
*TGFβ*	CCT-GAG-TGG-CTG-TCT-TTT-GA	CGT-GGA-GTT-TGT-TAT-CTT-TGC-TG
*TLR2*	CGT-TGT-TCC-CTG-TGT-TGC	AAA-GTG-GTT-GTC-GCC-TGC-T
*TLR4*	TTC-ACC-TCT-GCC-TTC-ACT-ACA	GGG-ACT-TCT-CAA-CCT-TCT-CAA

**Table 2:** Biological parameters characterizing obese *db*/*db* mice.

	*db*/*db* Mice	*db*/*db*^+^ Mice
Total body weight (g)	46.78 ± 3.39 **	26.56 ± 3.04
Subcutaneous adipose tissue (g)	1.03 ± 0.41 ***	0.10 ± 0.03
Visceral adipose tissue (g)	1.25 ± 0.53 **	0.46 ± 0.09
Liver (g)	2.31 ± 0.37 *	0.99 ± 0.27
Pancreas (g)	0.15 ± 0.05	0.18 ± 0.02
Heart (g)	0.17 ± 0.01	0.18 ± 0.03
Fasting glycemia (mg/dL)	595.80 ± 3.77 ***	346.20 ± 6.91

Data are expressed as means ± SEM (*n* = 10), * *p* < 0.05, ** *p* < 0.01, *** *p* < 0.005 as compared to *db*/*db*^+^ mice.

**Table 3:** Effect of *P. gingivalis* bacteria and LPS on the expression of genes encoding markers related to fibrosis in 3T3-L1 adipocytes.

Genes	Differentiated Adipocytes Exposed to *P. gingivalis* Bacteria and LPS for 48 h			Adipocytes Differentiated in the Presence of *P. gingivalis* Bacteria and LPS During 12 d		
	Control	Bacteria	LPS	Control	Bacteria	LPS
*TGFβ*	1.00 ± 0.02	1.51 ± 0.12 **	1.25 ± 0.03	1.00 ± 0.05	1.39 ± 0.12 *	1.02 ± 0.04
*FN1*	1.00 ± 0.06	1.53 ± 0.17 *	1.13 ± 0.14	1.00 ± 0.11	1.60 ± 0.07 **	1.35 ± 0.04 *
*Col1a1*	1.00 ± 0.11	1.21 ± 0.21	1.13 ± 0.23	1.00 ± 0.06	1.24 ± 0.11	1.03 ± 0.09
*Col3a1*	1.00 ± 0.09	1.11 ± 0.10	1.12 ± 0.19	1.00 ± 0.04	1.69 ± 0.25 *	1.50 ± 0.07

Differentiated adipocytes were exposed or not to *P. gingivalis* bacteria or LPS for 48 h, and adipocytes were differentiated in the presence or not of *P. gingivalis* bacteria or LPS for 12 d. The expression of the genes *TGFβ*, *FN1*, *Col1a1* and *Col3a1* was evaluated by RT-qPCR and normalized to *GAPDH* gene expression. Data were expressed as mean ± SEM of *n* = 4 independent experiments for the acute condition and *n* = 3 independent experiments for the chronic condition. * *p* < 0.05, ** *p* < 0.01 as compared to Control.

**Table 4:** Effect of *P. gingivalis* bacteria and LPS on the expression of genes encoding markers related to oxidative stress in 3T3-L1 adipocytes.

Genes	Differentiated Adipocytes Exposed to *P. gingivalis* Bacteria and LPS for 48 h			Adipocytes Differentiated in the Presence of *P. gingivalis* Bacteria and LPS During 12 d		
	Control	Bacteria	LPS	Control	Bacteria	LPS
*NOX2*	1.00 ± 0.07	1.81 ± 0.23 *	1.86 ± 0.26 *	1.00 ± 0.04	1.98 ± 0.16 **	1.55 ± 0.12 *
*NOX4*	1.00 ± 0.07	1.56 ± 0.09 **	1.59 ± 0.11 **	1.00 ± 0.09	2.37 ± 0.24 **	2.25 ± 0.28 *
*iNOS*	1.00 ± 0.05	1.49 ± 0.09 *	1.22 ± 0.20	1.00 ± 0.06	1.73 ± 0.19 *	0.93 ± 0.24
*GPx*	1.00 ± 0.04	1.15 ± 0.08	1.47 ± 0.19 *	1.00 ± 0.07	1.51 ± 0.03 *	1.92 ± 0.19 **
*Cu/ZnSOD*	1.00 ± 0.07	1.15 ± 0.13	1.02 ± 0.13	1.00 ± 0.04	1.37 ± 0.08 *	1.34 ± 0.06 *
*MnSOD*	1.00 ± 0.01	1.57 ± 0.11 **	1.71 ± 0.14 **	1.00 ± 0.04	1.67 ± 0.13 *	1.75 ± 0.17 *
*Catalase*	1.00 ± 0.01	1.58 ± 0.09 ***	1.35 ± 0.09 *	1.00 ± 0.07	1.96 ± 0.30 *	1.80 ± 0.12 *
*Nrf2*	1.00 ± 0.01	1.67 ± 0.06 *	1.94 ± 0.30 *	1.00 ± 0.08	1.20 ± 0.05 *	1.26 ± 0.06 *

Differentiated adipocytes were exposed or not to *P. gingivalis* bacteria or LPS for 48 h, and adipocytes were differentiated in the presence or not of *P. gingivalis* bacteria or LPS for 12 d. The expression of genes encoding redox markers was evaluated by RT-qPCR and normalized to *GAPDH* gene expression. Data were expressed as mean ± SEM of *n* = 4 independent experiments for the acute condition and *n* = 3 independent experiments for the chronic condition. * *p* < 0.05, ** *p* < 0.01, *** *p* < 0.005 as compared to Control.

**Table 5:** Effect of *P. gingivalis* bacteria and LPS on the expression of genes encoding markers related to adipogenesis and metabolic response in 3T3-L1 adipocytes.

Genes	Differentiated Adipocytes Exposed to *P. gingivalis* Bacteria and LPS for 48 h			Adipocytes Differentiated in the Presence of *P. gingivalis* Bacteria and LPS During 12 d		
	Control	Bacteria	LPS	Control	Bacteria	LPS
*C/EBPα*	1.00 ± 0.08	1.02 ± 0.10	0.90 ± 0.09	1.00 ± 0.06	1.33 ± 0.10 *	0.98 ± 0.05
*PPARγ*	1.00 ± 0.01	0.95 ± 0.08	0.90 ± 0.07	1.00 ± 0.08	1.09 ± 0.11	1.10 ± 0.08
*SREBP1c*	1.00 ± 0.03	1.25 ± 0.10	1.23 ± 0.19	1.00 ± 0.07	1.10 ± 0.10	0.97 ± 0.08
*FAS*	1.00 ± 0.04	1.23 ± 0.08	1.08 ± 0.14	1.00 ± 0.02	1.07 ± 0.11	0.87 ± 0.04
*LPL*	1.00 ± 0.05	1.22 ± 0.12	1.06 ± 0.08	1.00 ± 0.14	1.27 ± 0.06	1.09 ± 0.06
*ATGL*	1.00 ± 0.092	1.08 ± 0.19	1.06 ± 0.17	1.00 ± 0.07	1.76 ± 0.10 **	1.48 ± 0.11 *
*HSL*	1.00 ± 0.06	0.90 ± 0.16	1.02 ± 0.16	1.00 ± 0.09	1.01 ± 0.15	1.21 ± 0.09
*GLUT4*	1.00 ± 0.12	0.97 ± 0.10	0.96 ± 0.17	1.00 ± 0.09	1.37 ± 0.31	1.17 ± 0.27

Differentiated adipocytes were exposed or not to *P. gingivalis* bacteria or LPS for 48 h, and adipocytes were differentiated in the presence or not of *P. gingivalis* bacteria or LPS for 12 d. The expression of genes coding for adipogenesis and metabolic markers was assessed by RT-qPCR and normalized to *GAPDH* gene expression. Data were expressed as mean ± SEM of *n* = 4 independent experiments for the acute condition and *n* = 3 independent experiments for the chronic condition. * *p* < 0.05, ** *p* < 0.01 as compared to Control.

**Table 6:** Effect of polyphenols on the viability and the production of markers related to inflammatory status and oxidative stress in 3T3-L1 adipose cells exposed to *P. gingivalis* LPS.

Markers			LPS +		
	Control	LPS	Caffeic Acid	Quercetin	Epicatechin
Cell viability	100.00 ± 4.14	100.47 ± 6.10	103.07 ± 5.87	98.98 ± 3.44	101.71 ± 3.67
*TLR2*	1.00 ± 0.02	1.39 ± 0.04 ***	1.17 ± 0.03 ##	1.16 ± 0.05 ##	1.07 ± 0.08 ###
*TLR4*	1.00 ± 0.05	1.03 ± 0.04	0.89 ± 0.06	0.94 ± 0.07	0.90 ± 0.11
*NFκB*	1.00 ± 0.08	1.32 ± 0.04 **	1.04 ± 0.05 #	0.99 ± 0.04 ##	0.99 ± 0.11 #
*MyD88*	1.00 ± 0.03	1.24 ± 0.01 *	0.90 ± 0.02 ##	0.90 ± 0.01 ##	0.81 ± 0.04 ##
IL-6	9.61 ± 0.36	18.77 ± 2.13 **	14.53 ± 1.56	12.39 ± 1.03 #	12.03 ± 0.65 #
MCP-1	712.37 ± 69.22	1328.18 ± 113.99 ***	1097.03 ± 88.63	1044.45 ± 90.04	976.69 ± 58.07 #
Resistin	37.79 ± 0.082	46.54 ± 1.84 *	37.20 ± 1.73 #	37.39 ± 2.60 #	34.13 ± 0.92 ##
Leptin	17.69 ± 1.91	19.21 ± 2.87	14.25 ± 3.35	14.28 ± 2.62	16.24 ± 1.57
Adiponectin	3083.36 ± 104.68	2575.44 ± 93.56 *	2626.13 ± 194.13	2245.06 ± 230.11	2131.96 ± 158.57
ROS levels (3 h)	100.00 ± 3.13	119.44 ± 5.17 **	84.97 ± 2.80 ###	78.95 ± 2.61 ###	80.60 ± 3.81 ###
ROS levels (6 h)	100.00 ± 2.59	113.97 ± 2.49 *	89.98 ± 2.51 ###	80.57 ± 1.76 ###	83.72 ± 2.64 ###
ROS levels (48 h)	100.00 ± 3.79	107.09 ± 5.87	95.51 ± 5.02	92.42 ± 4.01 #	81.06 ± 1.85 ##
*NOX2*	1.00 ± 0.06	1.79 ± 0.12 *	1.13 ± 0.09 #	1.15 ± 0.10 #	1.21 ± 0.02 #
*NOX4*	1.00 ± 0.04	1.61 ± 0.10 ***	1.04 ± 0.06 ###	1.08 ± 0.04 ##	1.07 ± 0.10 ##
*iNOS*	1.00 ± 0.12	1.05 ± 0.16	1.19 ± 0.03	0.96 ± 0.28	1.25 ± 0.20
*GPx*	1.00 ± 0.09	1.34 ± 0.05 **	1.09 ± 0.04 #	1.08 ± 0.03 #	1.12 ± 0.07
*CuZnSOD*	1.00 ± 0.04	1.25 ± 0.02	1.14 ± 0.07	1.08 ± 0.07	1.27 ± 0.11
*MnSOD*	1.00 ± 0.03	1.31 ± 0.05 *	1.10 ± 0.06	1.00 ± 0.04 #	1.06 ± 0.11
*Catalase*	1.00 ± 0.07	0.98 ± 0.03	0.83 ± 0.05	0.84 ± 0.02	0.81 ± 0.04
*Nrf2*	1.00 ± 0.02	1.28 ± 0.06 **	1.04 ± 0.05 #	1.01 ± 0.07 #	0.96 ± 0.04 ##

Adipose cells were exposed to vehicle (Control) or *P. gingivalis* LPS (LPS) in the presence or not of caffeic acid, quercetin and epicatechin for 48 h, and their viability was assessed by MTT assay (% as compared to Control). Adipocytes were exposed to vehicle (Control) or *P. gingivalis* LPS (LPS) in the presence or not of polyphenols for 48 h or indicated time. The expression of genes coding for inflammatory and redox markers normalized to *GAPDH* gene expression, intracellular ROS levels (% as compared to Control) and secreted levels of adipokines (pg/mg proteins) were assessed by RT-qPCR, DCFH-DA assay and specific ELISA kits, respectively. Data were expressed as mean ± SEM of *n* = 4 independent experiments. * *p* < 0.05, ** *p* < 0.01, *** *p* < 0.005 as compared to Control; # *p* < 0.05, ## *p* < 0.01, ### *p* < 0.005 as compared to LPS.

## Data Availability

The original contributions presented in this study are included in the article. Further inquiries can be directed to the corresponding author.

## Abbreviations

The following abbreviations are used in this manuscript:

ATGL	Adipose triglyceride lipase
BCA	Bicinchoninic acid
CRP	C-reactive protein
C/EBPα	CCAAT enhancer binding protein alpha
Cu/ZnSOD	Copper-zinc superoxide dismutase
Col1a1	Collagen type I alpha 1 chain
Col3a1	Collagen type III alpha 1 chain
DCFH-DA	2′,7′-dichlorofluorescein diacetate
FAS	Fatty acid synthase
FN1	Fibronectin 1
GAPDH	Glyceraldehyde-3-phosphate dehydrogenase
GLUT4	Glucose transporter type 4
GPx	Glutathione peroxidase
HO-1	Heme oxygenase-1
HSL	Hormone-sensitive lipase
IL	Interleukin
iNOS	Inducible nitric oxide synthase
LDL	Low-density lipoprotein
LDLR	LDL receptor
LPL	Lipoprotein lipase
LPS	Lipopolysaccharides
MCP-1	Monocyte chemoattractant protein-1
MnSOD	Manganese-dependent superoxide dismutase
MyD88	Myeloid differentiation primary response 88
NFκB	Nuclear factor κappa B
NOX	NADPH oxidase
Nrf2	Nuclear factor erythroid 2-related factor 2
OMV	Outer membrane vesicles
PBS	Phosphate-buffer saline
PCSK9	Proprotein convertase subtilisin/kexin type 9
PGC-1α	Peroxisome proliferator-activated receptor-gamma coactivator-1alpha
PPARγ	Peroxisome proliferator-activated receptor
ROS	Reactive oxygen species
SOCS3	Suppressor of cytokine signaling 3
SREBP1c	Sterol regulatory element binding transcription factor 1c
T2DM	Type 2 diabetes mellitus
TGFβ	Transforming growth factor-β
TLR	Toll-like receptor
TNFα	Tumor necrosis factor-alpha
VLDL	Very-low-density lipoprotein
